# Research on the logical mechanism and path of digital new quality productivity enabling common prosperity

**DOI:** 10.1371/journal.pone.0354953

**Published:** 2026-08-05

**Authors:** Yujie Cui, Lin Li

**Affiliations:** College of Economic and Management, Heilongjiang Bayi Agricultural University, Daqing, Heilongjiang, China; Ningbo University, CHINA

## Abstract

Precisely measuring the phased achievements of common prosperity and analyzing the spatial distribution characteristics and driving mechanisms of its development level are crucial for promoting high-quality economic development. Based on panel data from 30 provincial regions in China, this study employs the entropy weight TOPSIS method, kernel density estimation, and dynamic qualitative comparative analysis (QCA) to explore the linkage effects and path selection of digital new quality productivity elements on the balanced development of common prosperity. The research findings are as follows: (1) The level of common prosperity in our country has generally exhibited an upward trend, and the spatial distribution pattern characterized by “higher levels in the east and lower levels in the west” is clearly evident. (2) The multi-dimensional elements of digital new quality productivity work in concert to promote the development of common prosperity, forming three configuration pathways: the “digital intelligence foundation - talent traction type,” “digital intelligence industry-talent linkage type,” and “digital intelligence development - talent collaboration type.” (3) Considering the evolving trend of the path toward common prosperity, the process has progressed through three distinct stages: “digital intelligence foundation,” “digital intelligence industry,” and “digital intelligence development.” Spatially, this evolution exhibits both an “East-middle disparity” and an “East-West disparity.” This research contributes to a better understanding of the current state of common prosperity, enhances the level of digital new quality productivity, and offers valuable insights for implementing differentiated development strategies to accelerate the reduction of regional economic disparities.

## 1. Introduction

Common prosperity, recognized as a key global development goal, aims to achieve balanced coordination among social, economic, and political dimensions, thereby narrowing the wealth gap and fostering fairness, justice, and sustainable development [1]. In the context of globalization, the concept of common prosperity aligns closely with the United Nations’ Sustainable Development Goals of “eradicating poverty” and “reducing inequality,” reflecting the universal aspiration to achieve these goals across all countries. China has established common prosperity as a central objective for achieving socialist modernization, emphasizing that it can be promoted through high-quality development. However, structural challenges, such as regional development imbalances, urban-rural income disparities, and insufficient economic growth momentum, pose significant obstacles to achieving common prosperity [2]. In the context of deep digital penetration and global digital transformation, leveraging new technologies to bridge the development gap between urban and rural areas and promote inclusive economic growth has become a critical issue for achieving common prosperity.

In the era of the digital economy, digital new quality productivity, as the core drivers of digital development, generate new opportunities for technology-driven economic growth and help address challenges associated with achieving common prosperity. This study conceptualizes “digital new quality productivity” as an advanced production paradigm governed by the logic of a “technology-economic paradigm.” Diverging from views that consider digital technologies as merely one-dimensional tools, digital new quality productivity underscores the coordinated development of multiple dimensions, such as digital infrastructure, digital talent, and emerging industries. This multifaceted synergy, achieved via the digital reconfiguration of labor, labor inputs, and production objects, modifies traditional input-output relationships in established production functions, resulting in transformative advancements in value-creation mechanisms and economic growth paradigms [3]. Previous research has investigated the effects of digital talent, the digital economy, or innovation systems on income distribution and social welfare [4–6]. Other studies have also examined the influence of digital new quality productivity on economic development and sustainable transformation [7,8]. Nevertheless, the majority of existing analyses emphasize isolated factors or utilize static data, constraining the comprehension of the collaborative evolution among these multi-dimensional components and their synergistic effects on common prosperity [9–11]. Against this backdrop, this paper analyzes the mechanisms through which digital new quality productivity affects common prosperity and proposes relevant policy measures, thereby providing theoretical support for understanding inclusive growth within the digital era.

The remaining sections of this article are organized as follows: Section 2 reviews the relevant literature and presents the research framework. Section 3 outlines the research design. Section 4 analyzes the development status and spatio-temporal evolution results of common prosperity. Section 5 analyzes the configuration results of digital new quality productivity, empowering common prosperity. Section 6 discusses the results. Section 7 presents the research conclusions, policy implications, the limitations, and prospects.

## 2. Literature review and research framework

### 2.1. Literature review

Internationally, research on digital new quality productivity and common prosperity primarily focuses on three aspects:

First, the theoretical foundations of digital new quality productivity. The international academic community has not directly adopted the concept of “digital new quality productivity.” Nevertheless, related research has delved into the integration of its elements and the transformational impetus from perspectives such as the technological-economic paradigm, innovation-driven growth, and the production effects of digital technologies. The technological-economic paradigm theory posits that technological revolutions induce changes in economic forms and institutional structures by redefining production factors and methods [12]. In the era of the digital economy, the innovation and application of digital technologies have given rise to new “technology-economic paradigms.” These paradigms, by altering production methods, organizational structures, and value-creation mechanisms, drive industrial structure reform and qualitative changes in productivity [13,14]. Meanwhile, the theory of innovation-driven growth posits that knowledge spillovers, human capital mobility, and regional innovation capabilities constitute the core mechanisms driving productivity enhancement [15]. These factors, acting in concert, promote technological and industrial innovation, which in turn drives the qualitative transformation of productivity. Furthermore, research on digital capabilities, the platform economy, and network effects suggests that integrating information resources and data-driven collaborative innovation can significantly enhance production efficiency and value-creation capabilities [16–18]. It is evident that these studies have preliminarily explored how digital technological innovation drives productivity transformation, promotes industrial upgrading, and contributes to social and economic development, thereby laying a theoretical foundation for the development of digital new quality productivity. This article introduces the concept of “digital new quality productivity,” aiming to integrate digital technologies, human capital, and mechanisms of industrial transformation. This integration provides important support for understanding the leap in productive forces in the digital era and its impacts on social development, income distribution, and common prosperity.

Second, research on the development of common prosperity. Scholars generally view common prosperity as a long-term, complex process, with discussions centering on its conceptual definition, measurement, and evaluation, and driving factors. Conceptually, common prosperity originates in the Marxist theory of social productive forces, which emphasizes the quality of productive forces and the characteristics of production relations [19]. It represents a historical outcome of the development of productive forces and social progress. The academic community typically defines common prosperity from two dimensions: “common” and “wealth.” Here, the term “common” denotes the inclusiveness of economic development achievements, aiming to promote social equity and sustainable development by narrowing development gaps between urban and rural areas, among regions, and across different groups [20]. “Prosperity” emphasizes the harmonious development of material prosperity and spiritual life, encompassing economic growth, income distribution, public services, and the environment [21,22]. In terms of measurement and evaluation, most scholars employ a quantifiable indicator system and a quantitative research paradigm [23,24]. For instance, Zhang et al. developed an evaluation system for common prosperity based on seven dimensions: income gap, economic level, cultural and entertainment activities, infrastructure, urbanization rate, life expectancy, and employment rate. They employed the probabilistic TOPSIS model with ordered weighted distance and entropy weight to evaluate the level of common prosperity in Zhejiang Province [25]. Some scholars have also found that the level of common prosperity in China has been steadily increasing. However, an evident “high in the east and low in the west” spatial pattern persists. These regional disparities are the primary cause of the imbalance in spatial development [26,27]. In terms of driving factors, scholars have explored the mechanisms through which various factors contribute to common prosperity from perspectives such as rural development [28], labor mobility [29], the digital economy [30], and low-carbon transformation [31]. Some scholars have also conducted in-depth analyses of the driving mechanisms and value-creation pathways of common prosperity, considering multiple factors such as green finance and ecological environmental protection [32].

Third, the mechanism by which digital new quality productivity drives common prosperity. Current research has not explored the relationship between digital new quality productivity and common prosperity; however, relevant literature provides useful insights into the underlying mechanisms. Based on dimensions such as digital productivity, digital inclusive finance, artificial intelligence technology, and the digital economy, scholars have preliminarily explored how specific components of the digital new quality productivity reshape employment structure [33], promote inclusive growth [34], improve income distribution [35], and drive sustainable development [36]. However, this research paradigm, which relies solely on the “net effect” of a single factor, has methodological limitations. Such approaches typically simplify complex driving factors into independent variables and evaluate their marginal effects separately, thereby overlooking the interrelationships and interactions among the internal elements of digital new quality productivity. Therefore, some scholars have employed the QCA method, shifting the research focus from the impact of a single variable to the identification of multiple configurational pathways that lead to the same outcome. Currently, the QCA method has been widely applied in social science research areas such as technological innovation [37], high-quality regional economic development [38], and green and low-carbon transformation [39]. However, it has limitations in causal studies examining the relationship between digital new quality productivity and common prosperity. The impact of digital new quality productivity on common prosperity is inherently dynamic and continuous. Traditional QCA methods based on static cross-sectional data are unable to capture the dynamic relationships and spatiotemporal interactions among complex factors.

Overall, the existing research identifies three research gaps. (1) The existing literature has scarcely examined the relationship between digital new quality productivity and common prosperity. Although relevant studies have established frameworks for digital new quality productivity, these frameworks primarily emphasise the conventional dimensions of productivity evolution driven by technological innovation. (2) Empirical studies on common prosperity frequently rely on traditional regression methods to examine the linear impact of individual factors, yet they overlook the configurational effects resulting from combinations of multiple factors. (3) Existing QCA studies primarily focus on the static relationships between antecedents and outcomes, while overlooking the temporal effects of digital new quality productivity on common prosperity and the associated regional heterogeneity. The primary contributions of this paper are as follows: First, by integrating the multidimensional features of digital new quality productivity, we propose a theoretical model comprising “digital labourer–object of digital labour–digital means of labour,” which systematically elucidates the complex mechanism through which digital new quality productivity affects common prosperity. Second, using entropy-weighted TOPSIS and kernel density estimation, we comprehensively evaluate the development status and spatiotemporal characteristics of common prosperity, thereby enhancing the precision of the analysis. Finally, the dynamic QCA method, we identify the configurational pathways by which digital new quality productivity drives common prosperity and reveal the temporal evolution and spatial heterogeneity of each pathway, offering implications for policy-making.

### 2.2. Research framework

Digital new quality productivity consists of several elements. These include technological innovation, industrial upgrading, and human capital. Its impact on common prosperity is multi-dimensional, non-linear, and complex. Therefore, it is necessary to analyze the interrelationships and driving pathways of these elements from a systemic perspective to understand how digital new quality productivity promotes common prosperity.

This paper constructs a research framework based on the technology-economic paradigm, complex systems theory, and productivity theory. The technology-economic paradigm posits that the collaborative evolution of technological innovation, institutional change, and social structures can reshape the economic system and promote sustainable social prosperity [40]. In the era of the digital economy, the deep integration and extensive penetration of big data, artificial intelligence, and other digital technologies, together with the growing importance of data as a production factor, have spurred the emergence of a new technology-economic paradigm centered on digital new quality productivity. This paradigm not only reconfigures the logic of factor allocation and value creation but also drives systemic changes and technological transformation across various sectors of the economy and society. The theory of complex systems posits that interactions among diverse elements within a socio-economic system can enhance overall system efficiency [41]. Therefore, the impact of digital new quality productivity on common prosperity can be viewed as an emergent effect arising from the synergistic interactions among internal elements within the system. This viewpoint is highly consistent with the “multiple concurrent causes” and “different paths leading to the same destination” logic emphasized by the Dynamic QCA method, and it offers nonlinear, multi-path causal explanations. Based on productivity theory, digital new quality productivity represents an advanced mode of production. Its internal structure should be explained in terms of the three fundamental elements of productivity. Against the backdrop of technological innovation, digital and intelligent technologies have been profoundly integrated into the production process, leading to the reconfiguration of the connotations and relationships among laborers, labor objects, and labor materials [42]. Furthermore, through coordinated factor allocation, it reshapes the pattern of economic growth and social distribution. Therefore, analyzing the interaction mechanism among digital labourers, the object of digital labour and digital means of labour from a productivity system perspective is crucial for revealing the internal logic through which digital new quality productivity affects common prosperity.

**Fig 1:**
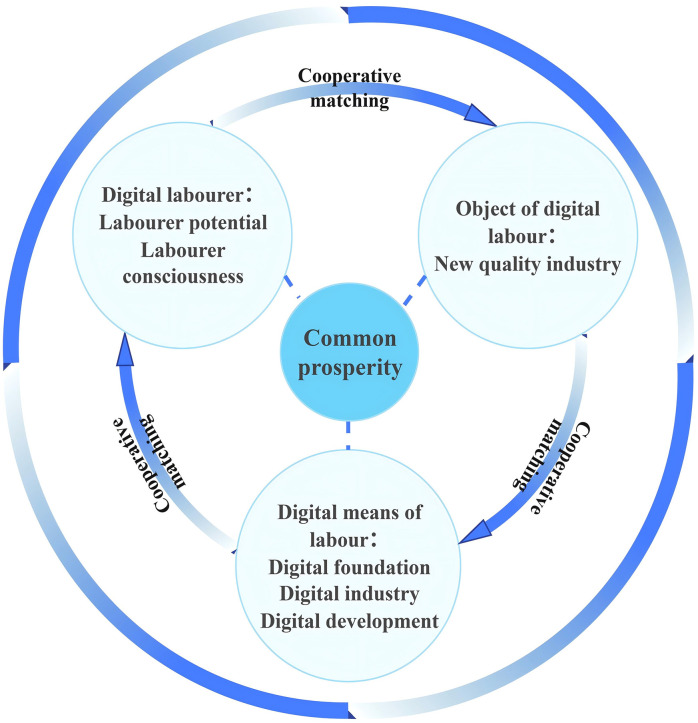
Research framework of digital new quality productivity enpowering common prosperity.

Based on the above theoretical analysis, this study constructs a theoretical framework comprising “digital labourer, object of digital labour and digital means of labour” (Fig 1). The framework aims to investigate how internal antecedent conditions within the digital new quality productivity system interact and jointly shape the development of common prosperity.

## 3. Research design

### 3.1. Variable construction

#### 3.1.1. Outcome variable: Common prosperity.

Based on the connotation of common prosperity and existing research [43–46], this paper constructs a common prosperity indicator system from five dimensions: governance capacity, economic prosperity, people’s wellbeing, spiritual prosperity, and ecological and environmental protection. Specifically, governance capacity is manifested through fiscal expenditures and investments in public services, which reflect the public orientation of governmental resource allocation. Meanwhile, the level of urbanization is employed to gauge the structural pressures confronted by social governance. Economic prosperity encompasses dimensions such as economic efficiency, income security, and industrial development, thereby reflecting the accumulation of material wealth, the quality of income distribution, and the extent of industrial structural upgrading. The core of people’s well-being lies in the equitable allocation of resources across sectors such as healthcare, transportation, social security, and educational investment, as well as in the upgrading of residents’ consumption structures. Spiritual prosperity emphasizes the all-round development of individuals, with the level of social civilization being measured by the supply of cultural resources and residents’ cultural consumption levels. Ecological and environmental protection focuses on the sustainability of development, and its effectiveness in promoting ecological civilization can be reflected in ecological construction and pollution control efforts. Detailed indicator explanations are shown in Table 1.

**Table 1:** Evaluation system for the common prosperity index.

Subsystem level	Criterion layer	Indicator layer
Governance ability	Local fiscal expenditure	Local fiscal expenditure/GDP
	Public service	The proportion of local expenditure on public services
	Urbanization rate	Urban population/Year-end permanent population
Economic prosperity	Economic efficiency	Gross domestic product per capita
		Per capita years of schooling * Employed people in urban units
	Income security	Urban registered unemployment rate
		Per capita disposable income
	Industrial development	The value added of the tertiary industry/ GDP
		Total imports by place of business registration/ GDP
People’s wellbeing	Medical treatment and public health	Number of beds in medical and health institutions
		Number of health technicians per 10,000 population
	Transportation	Public transport vehicles per 10,000 people
		Highway mileage
	Social insurance	Local fiscal expenditure on social security and employment/GDP
		Number of participants in the basic old-age insurance for urban and rural residents
	Degree of education	School attendance per 100,000 persons in higher education
		Intensity of education expenditure
	Engel coefficient	Engel coefficient
Spiritual prosperity	Cultural quality	Public library holdings per capita
	Cultural development	Per capita cultural and entertainment consumption expenditure
Ecological and environmental protection	Green and environmental protection	Forest cover percentage
		Per capita green park area
		Rate of harmless treatment of domestic waste
	Pollution consumption	Energy utilization per unit of Gross Domestic Product
		Environmental pollution index

#### 3.1.2. Conditional variables: digital new quality productivity.

This paper refers to the research of Gao [47], focusing on digital labourer, object of digital labour and digital means of labour as the main directions. It selects 17 detailed variable indicators from 7 antecedent variables, including labourer potential, labourer consciousness, new quality industries, digital foundation, digital industry, and digital development. Detailed explanations of the indicators are provided in Table 2.

**Table 2:** Digital new quality productivity antecedent variable index assessment system.

Variable dimension	Antecedent variable	Variable representation
Digital labourer	Labourer potential	Information technology service personnel/total number
		Per capita salary of information technology service personnel
	Labourer consciousness	Tertiary industry employment/total employment
		Entrepreneurial activity level
Oobject of digital labour	New quality industry	Number of artificial intelligence patents
		Industrial robot installation density
Digital means of labour	Digital foundation	Fiber optic cable route length per square kilometer
		Number of internet broadband access subscribers
		Mobile phone base station density
	Digital industry	Number of informationized enterprises
		Number of websites per hundred companies
		Percentage of enterprises engaged in e-commerce transactions
		E-commerce transaction volume
	Digital development	Digital economy index
		Digital financial inclusion index
		Per capita patent

(1) Labourer potential: It reflects the digital skills level, knowledge reserves, and innovation capabilities of the workforce in economic activities, as well as the effects it has on promoting the accumulation of social wealth and the equitable distribution of income [48]. It is the key intellectual capital that drives the development of common prosperity. We represent it as the sum of the entropy values for the proportion of information technology service personnel and the per capita salary of information technology service personnel.
(2) Labourer consciousness: It can enhance workers’ employment initiative and entrepreneurial spirit, increase their income and living standards, and reduce the wealth gap among different social groups [49]. We represent it as the sum of the entropy values for the proportion of tertiary industry employment and the entrepreneurial activity level.
(3) New quality industry: According to Marshall’s theory of industrial agglomeration, industrial clustering effectively optimizes resource allocation, reduces industry-wide production costs, and enhances labor productivity by leveraging the labor-pool effect, knowledge spillover effect, and technology diffusion effect [50]. A new quality industry, leveraging the positive externalities of agglomeration in artificial intelligence, drives the upgrading of industrial models and a leap in social productivity, accelerates the transformation of the economic development mode, and provides the core driving force for achieving common prosperity. We represent it as the sum of the entropy values for the number of artificial intelligence patents and the industrial robot installation density for representation.
(4) Digital foundation: The cornerstone of common prosperity. It achieves an efficient flow of resources and factors across regions and between urban and rural areas by strengthening regional exchanges and cooperation, laying the foundation for coordinated regional development and the realization of common prosperity [51]. We represent it as the sum of the entropy values for the fiber-optic cable route length per square kilometer, the number of internet broadband access subscribers, and mobile phone base station density.
(5) Digital industry: The core value of the digital industry lies in driving the transformation and upgrading of traditional industries, expanding their scale, thereby creating employment opportunities, increasing residents’ income, and providing a solid foundation for common prosperity. We represent it using the sum of the number of informationized enterprises, the number of websites per hundred companies, the percentage of enterprises engaged in e-commerce transactions, and the entropy value of e-commerce transaction volume.
(6) Digital development: Reduces information asymmetry and market transaction costs, thereby optimizing the efficiency of social resource allocation [52]. Additionally, it expands residents’ income channels through the penetration of digital technology and the inclusiveness of digital finance, promotes economic equality, and accelerates the coordinated development of common prosperity across regions [53]. We represent it as the sum of the entropy values for the digital economy index, the digital financial inclusion index, and the per capita patent.

### 3.2. Methods of research

This paper integrates multi-criteria decision-making evaluation, non-parametric statistical methods, and qualitative comparative analysis to assess the development level of common prosperity and to explore its configuration-driven pathways. Firstly, the entropy-weight TOPSIS method performs a multi-dimensional comprehensive evaluation to ensure the objectivity of indicator weights, thereby providing a reliable quantitative basis for subsequent empirical analysis [54]. Secondly, based on the estimation results, kernel density estimation is further employed to examine the distribution patterns and polarization trends of common prosperity, as well as to identify provincial disparities and dynamic evolutionary characteristics. Finally, in response to the temporal and spatial heterogeneity of common prosperity, the dynamic QCA method is employed to identify key configurational conditions and to reveal the complex causal mechanisms as well as the cross-temporal evolutionary patterns through which digital new quality productivity affect common prosperity. This methodological integration establishes an analytical chain from “static measurement” to “dynamic evolution” and further to “cross-temporal and spatial causal mechanisms,” which not only accurately captures the current development status and regional disparities of common prosperity, but also uncovers its underlying driving forces, thereby providing a new analytical perspective for research on common prosperity development.

#### 3.2.1. Entropy-weighted TOPSIS model.

The entropy weight TOPSIS method is typically used for multi-objective evaluation and decision-making. It ranks the superiority and inferiority of decision-making evaluations by calculating the degree of fit between the evaluation targets and the “optimal solution scheme” [55]. It combines the advantages of the entropy weight method and the TOPSIS method for analysis, providing decision-makers with scientific, objective, and comprehensive decision-making analysis, and to a certain extent, improving the accuracy of index analysis and data processing [56]. Therefore, this paper introduces the entropy-weighted TOPSIS model to precisely measure the level of common prosperity in China from 2013 to 2022. The specific formula steps are as follows.

The first step is the standardization of the initial indicators (set as $x_{ij}$ the initial evaluation value, among i=1,2,3,⋯,m, j=1,2,3,⋯,n, where m is the number of evaluation indicators, and n is the number of evaluation indicators). The precise calculation is detailed below.

Standardization of positive indicators:


$$\begin{array}{c} x_{ij}^{\prime }=\frac{x_{ij}-min\left(x_{j}\right)}{max\left(x_{j}\right)-min\left(x_{j}\right)} \end{array}$$ (1)

Negative indicator standardization:


$$\begin{array}{c} x_{ij}^{\prime }=\frac{max\left(x_{j}\right)-x_{ij}}{max\left(x_{j}\right)-min\left(x_{j}\right)} \end{array}$$ (2)

The second step involves calculating the proportion $P_{ij}$ of item j in year i.


$$\begin{array}{c} P_{ij}=\frac{x_{ij}^{\prime }}{\sum a\sum_{i}x_{ij}^{\prime }} \end{array}$$ (3)

The third step is to calculate the information entropy of the j index, where $k=\frac{\text{1}}{ln(mn)}$ and K>0, making $e_{j}$ >0.


$$\begin{array}{c} e_{j}=-k\sum_{i}P_{ij}ln\left(P_{ij}\right) \end{array}$$ (4)

The fourth step is to calculate the redundancy of information entropy of item j.


$$\begin{array}{c} d_{j}=\text{1}-e_{j} \end{array}$$ (5)

The fifth step is to calculate the weight of item j indicator.


$$\begin{array}{c} W_{j}=\frac{d_{j}}{\sum_{j}d_{j}} \end{array}$$ (6)

The sixth step is to calculate the entropy of the index.


$$\begin{array}{c} S=w_{j}\times x_{ij}^{\prime } \end{array}$$ (7)

The seventh step is to build the weighted decision matrix.


$$\begin{array}{c} x_{ij}=x_{ij}^{\prime }\times w_{j} \end{array}$$ (8)

The eighth step is to calculate the Euclidean distance.


$$\begin{array}{c} D_{i}^{+}=\sqrt{\sum ({Z_{ij}-Z_{j}^{*+})}^{\text{2}}} \end{array}$$ (9)

$$\begin{array}{c} D_{i}^{-}=\sqrt{\sum ({Z_{ij}-Z_{j}^{*-})}^{\text{2}}} \end{array}$$ (10)

The ninth step is to calculate the relative fit between the index and the optimal solution.


$$\begin{array}{c} C_{i}=\frac{D^{-}}{D_{i}^{+}+D_{i}^{-}} \end{array}$$ (11)

#### 3.2.2. Kernel density estimation.

This paper uses kernel density estimation to comprehensively grasp the spatio-temporal distribution and dynamic evolution of the development level of common prosperity across the country and in various regions. Kernel density estimation is a non-parametric method that uses a continuous probability density function to depict the evolution of a random variable, capable of objectively and accurately capturing the distribution of data [57,58]. Its calculation formula is as follows:


$$\begin{array}{c} f(x)=\frac{\text{1}}{Nh}\sum_{i=\text{1}}^{N}K(\frac{x_{i}-\overset{―}{x}}{h}) \end{array}$$ (12)

$$\begin{array}{c} K(x)=\frac{\text{1}}{\sqrt{\text{2}\pi }}exp\left(-\frac{x^{\text{2}}}{\text{2}}\right) \end{array}$$ (13)

Here, f(x) is the density function, and N represents the number of observed samples, $k_{\left(x\right)}$ is the Gaussian kernel function, $x_{i}$ are the independently distributed sample values, $\overset{―}{\text{x}}$ represents the sample mean, and h represents the bandwidth.

#### 3.2.3. Dynamic QCA method.

The Qualitative Comparative Analysis (QCA) method has clear advantages in handling multiple concurrent causal phenomena. However, traditional QCA relies mostly on static cross-sectional data and often ignores the time dimension, which can lead to errors in time selection and less robust results [59]. To address these limitations from “time omission,” Roberto Garcia-Castro introduced the dynamic panel QCA method. This method, based on the R language, compensates for static QCA’s lack of time and spatial analysis. Unlike static QCA, dynamic QCA measures consistency at three levels inter-group, intra-group, and summary and tracks subtle changes in the spatiotemporal dimension by adjusting the distance metric. This reveals how configuration paths differ over time [60]. Since digital new quality productivity affects common prosperity in a continuous process over time, traditional QCA struggles to capture configuration changes across time points. Therefore, this study uses dynamic QCA to show how common prosperity evolves under different institutional configurations and to reveal the dynamic effects of digital new quality productivity on its path.

### 3.3. Data source

Given the comparability and availability of the data, this study uses the 30 provincial regions of China from 2013 to 2022 as the research subjects (excluding Tibet, Hong Kong, Macao, and Taiwan). The sample covers 300 cases, and the data mainly come from authoritative reports such as the China Statistical Yearbook, the China Population and Employment Statistical Yearbook, the China Information Yearbook, the Information Industry Yearbook, the China Industrial Statistics Yearbook, the China Energy Statistical Yearbook, and the National Bureau of Statistics.

### 3.4. Data calibration

This paper uses the direct calibration method to uniformly standardize and calibrate the variables, and sets 75%, 50%, and 25% as the points of complete membership, intersection, and complete disaffiliation, respectively. To prevent the calibrated data from being excluded from the identification process, the value 0.5 has been uniformly changed to 0.501. The detailed calibration table is shown in Table 3.

**Table 3:** Data anchor analysis.

Variable			Calibrate analytical anchor points		
			Complete affiliation	Intersection	Complete disaffiliation
Condition variables	Digital labourer	Labourer potential (A)	0.018	0.012	0.008
		Labourer consciousness (B)	0.018	0.013	0.009
	Object of digital labour	New quality industry (C)	0.023	0.009	0.003
	Means of digital labour	Digital foundation (D)	0.035	0.022	0.014
		Digital industry (E)	0.040	0.021	0.013
		Digital development (F)	0.040	0.028	0.017
Outcome variable	Common prosperity (Y)		0.328	0.295	0.263

## 4. Development analysis of common prosperity

### 4.1. Measurement and analysis of common prosperity

**Fig 2:**
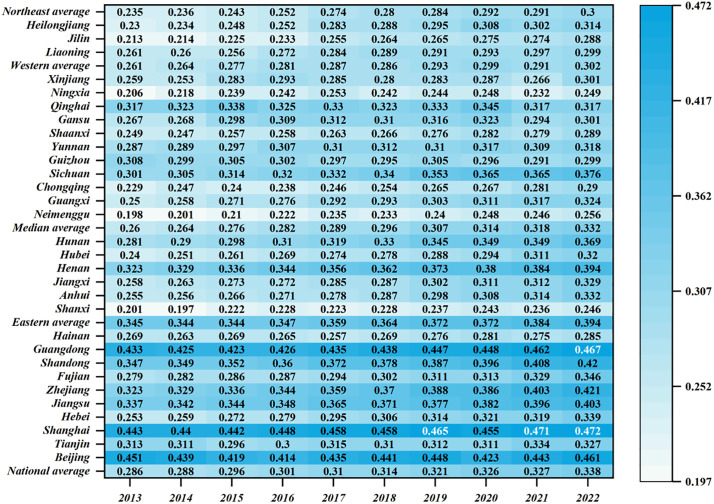
Measurement results of common prosperity.

Based on the aforementioned indicators for achieving common prosperity, this paper uses the entropy weight TOPSIS method to calculate the overall level of common prosperity in China. Based on the east, central region, west, and northeast, the differences in common prosperity among the regions were analyzed. The detailed calculation results are shown in Fig 2.

Overall, the level of common prosperity in our country has been increasing year by year. The national index of common prosperity has risen from 0.286 to 0.338, with an average annual growth rate of 1.68%. This might be attributed to our country’s long-term strategic policy of focusing on economic development. Since the 18th National Congress of the Communist Party of China, when common prosperity was recognized as the fundamental principle of socialism with Chinese characteristics, a series of policies, including targeted poverty alleviation, rural revitalization, and the digital economy, have been implemented in succession. These policies have effectively promoted coordinated development across science and technology, people’s livelihoods, the economy, culture, and ecology, and have significantly contributed to the achievement of common prosperity [61].

At the regional level, the level of common prosperity in each region shows a converging trend with the national level. Among them, the eastern region has the highest level of development, followed by the central, western, and northeastern regions in descending order. Specifically, Beijing, Shanghai, and Guangdong, as the “main battlefields” of economic development, play a significant “radiation role” in the process of common prosperity, driving the high-quality improvement of common prosperity in neighboring provinces. Zhejiang, Shandong, and Jiangsu provinces are in the second tier, forming the “main force” for the development of common prosperity. Henan, Sichuan, Hunan, Tianjin, and Qinghai have formed the backbone of the common prosperity development, while the construction of common prosperity in the remaining provinces shows a trend of catching up. The cross-sectional results from 2013 to 2022 show that the level of common prosperity across provinces has fluctuated slightly, and the regional gap coefficient has gradually decreased. Among them, there are significant internal differences in the eastern region, whereas those in the central and western regions are relatively small. The formation of this spatial pattern may be attributed to factors such as geographical location, policy support, and resource allocation. As most of the eastern provinces are coastal, they have gained a leading position in economic development, poverty alleviation, and narrowing the gap between urban and rural areas. Therefore, common prosperity is in the lead. However, the central and western regions are constrained by their inland geographical location and low openness. Despite the country’s efforts to allocate resources and implement industrial support policies, these resources and policies tend to concentrate in provincial capitals, further intensifying the “siphoning effect” and exacerbating regional development imbalances and reducing the level of common prosperity.

It is worth noting that although the overall and regional development towards common prosperity is showing a steady upward trend, the overall situation of common prosperity in our country is still at a relatively rudimentary stage, and there is a significant disparity in development among different regions. The current development stage indicates that there is still considerable room for improvement in the construction of common prosperity in our country. Each province should focus on high-quality development and closing regional gaps to promote common prosperity.

### 4.2. Analysis of the spatio-temporal evolution of common prosperity

**Fig 3:**
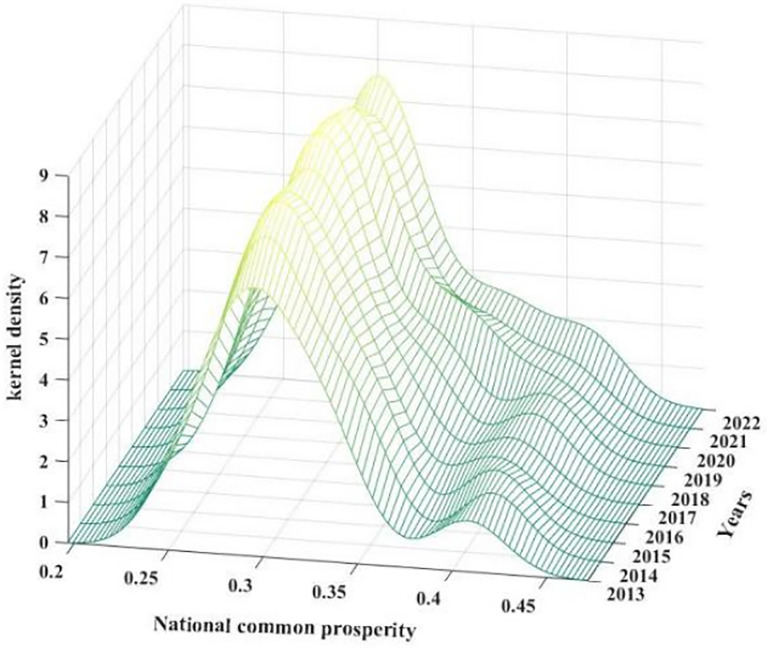
Nuclear density of national common prosperity.

**Fig 4:**
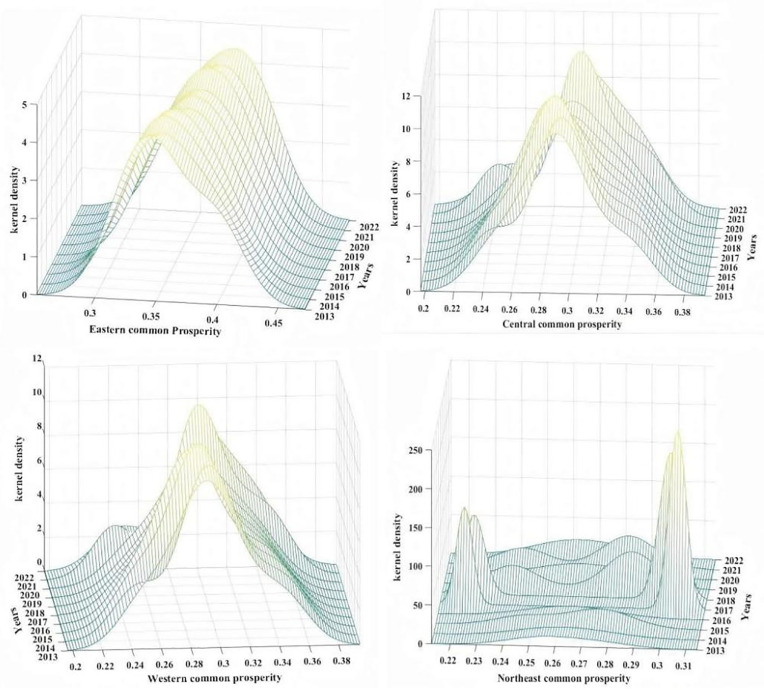
Nuclear density of regional common prosperity.

To explore the temporal and spatial variation in common prosperity across our country, this paper uses MATLAB to generate three-dimensional kernel density curves for the entire country and its regions, as shown in Figs 3 and 4.

As shown in Fig 3, the national kernel density curve shows a gradual rightward shift. This indicates that the overall level of common prosperity in our country has improved. It also shows that progress toward common prosperity has been positive. From the perspective of the distribution pattern, the kernel density curve shows a “rise-fall-rise” trend. The peak height increases while the peak width narrows. This suggests that the absolute gap and the trend of fluctuation in common prosperity in our country are gradually narrowing. From the perspective of distribution expansibility, the density curve of common prosperity from 2013 to 2019 showed a “double peak” pattern with a “right-tailing” phenomenon. After 2019, it shifted to a “single peak” form. This indicates that, over time, the polarization phenomenon of common prosperity in China has weakened. Common prosperity now shows a trend of more balanced development.

Fig 4 shows that the kernel density curves of each region gradually shift to the right. This indicates that the dynamic level of common prosperity development across regions is increasing. Common prosperity development in the eastern region is at the forefront nationwide. In contrast, the construction of common prosperity in the northeastern region is relatively lagging behind. This is in good agreement with the results obtained by the entropy weight TOPSIS method. Specifically, in the eastern region, the peak change trend is not significant, and there is no tailing phenomenon. This shows that the level of common prosperity in the region is relatively close and that there are no significant differences in development. The nuclear density curve in the central region shows a “rising first, then falling” trend, with 2015 as the turning point. After 2020, it has shown a slight recovery. This indicates that the development momentum in the central region is strong and that there is a clear upward trend. This may be closely related to the allocation of policy resources, the empowerment of science and technology, and the adjustment of the industrial structure. Since 2013, the peak values in the western region have been gradually increasing. By 2022, the peak value was reached. This indicates that the disparity in common prosperity in the western region has gradually narrowed, and the construction of common prosperity has shown a catching-up trend. The overall trend of the nuclear density peak in the northeastern region shows no clear pattern, and there is left-right tailing. This indicates that polarization of common prosperity in the northeastern region is quite significant. Overall, the level of common prosperity in our country exhibits the development characteristics of “leadership in the east, rise in the central region, development in the west, and revitalization in the northeast”.

## 5. Dynamic QCA analysis

### 5.1. Necessity analysis

The necessity test of antecedent conditions should be completed before configuration analysis. In this context, the necessary conditions for Dynamic QCA require the fulfillment of the following two criteria. The consistency level of an individual condition surpasses 0.9. The consistency adjustment distance is below 0.2. When the consistency adjustment distance for a prerequisite condition exceeds 0.2, further detailed analysis of the specific case data is needed [62]. Based on this theoretical framework, we use the statistical software R to carry out a dynamic QCA necessity analysis and present the necessary conditions in Table 4.

**Table 4:** Necessity analysis of individual conditions.

Variable	High level of common prosperity(Y)				Low level of common prosperity(~Y)			
	Aggregated consistency	Aggregated coverage	Inter-Group consistency distance	Intra-Group consistency distance	Aggregated consistency	Aggregated coverage	Inter-Group consistency distance	Intra-Group consistency distance
A	0.758	0.779	0.127	0.391	0.321	0.328	0.683	0.834
**~**A	0.346	0.339	0.312	0.776	0.784	0.763	0.272	0.299
B	0.705	0.722	0.302	0.408	0.372	0.379	0.719	0.805
~B	0.394	0.387	0.432	0.719	0.727	0.710	0.447	0.357
C	0.817	0.844	0.087	0.443	0.270	0.278	0.549	0.822
~C	0.301	0.293	0.360	0.868	0.848	0.821	0.142	0.207
D	0.848	0.851	0.047	0.357	0.280	0.279	0.650	0.794
~D	0.281	0.282	0.247	0.880	0.850	0.847	0.142	0.265
E	0.792	0.805	0.131	0.495	0.321	0.324	0.356	0.765
~E	0.335	0.332	0.331	0.822	0.807	0.795	0.073	0.431
F	0.784	0.787	0.443	0.196	0.321	0.320	0.897	0.765
~F	0.322	0.323	0.719	0.575	0.786	0.783	0.570	0.259

Note: ~ indicates not.

As shown in Table 4, during the development of both high and non-high common prosperity, the overall consistency of the antecedent conditions was below 0.9 in each case. Therefore, it can be preliminarily concluded that there are no necessary conditions that affect the development of high and low common prosperity. However, the inter-group consistency adjustment distance for labourer consciousness, new quality industry, digital foundation, digital industry, and digital development is greater than 0.2, which warrants further analysis. Table 5 shows the detailed results.

**Table 5:** Adjust inter-group data with distances exceeding 0.2.

Antecedent condition		Years									
		2013	2014	2015	2016	2017	2018	2019	2020	2021	2022
B-Y	Consistency between groups	0.369	0.438	0.475	0.521	0.610	0.700	0.731	0.785	0.860	0.836
	Coverage between groups	0.557	0.549	0.565	0.594	0.661	0.668	0.731	0.769	0.783	0.837
F-Y	Consistency between groups	0.274	0.346	0.531	0.482	0.535	0.771	0.844	0.957	0.997	0.993
	Coverage between groups	0.809	0.831	0.707	0.703	0.799	0.755	0.829	0.783	0.742	0.851
~A-Y	Consistency between groups	0.406	0.407	0.402	0.424	0.472	0.482	0.437	0.324	0.225	0.153
	Coverage between groups	0.093	0.113	0.158	0.233	0.396	0.534	0.725	0.729	0.696	0.796
~C-Y	Consistency between groups	0.558	0.389	0.362	0.393	0.397	0.373	0.316	0.245	0.163	0.213
	Coverage between groups	0.124	0.105	0.139	0.232	0.389	0.469	0.576	0.566	0.414	0.602
~D-Y	Consistency between groups	0.266	0.246	0.306	0.365	0.385	0.353	0.309	0.276	0.195	0.2
	Coverage between groups	0.062	0.067	0.116	0.212	0.362	0.480	0.59	0.633	0.541	0.704
~E-Y	Consistency between groups	0.168	0.159	0.273	0.325	0.413	0.426	0.404	0.356	0.279	0.312
	Coverage between groups	0.043	0.051	0.129	0.229	0.395	0.482	0.602	0.626	0.55	0.735
~F-Y	Consistency between groups	0.802	0.734	0.589	0.641	0.626	0.446	0.318	0.133	0.029	0.049
	Coverage between groups	0.164	0.172	0.198	0.298	0.447	0.561	0.716	0.813	0.926	1.000

Note: ~ indicates not.

**Fig 5:**
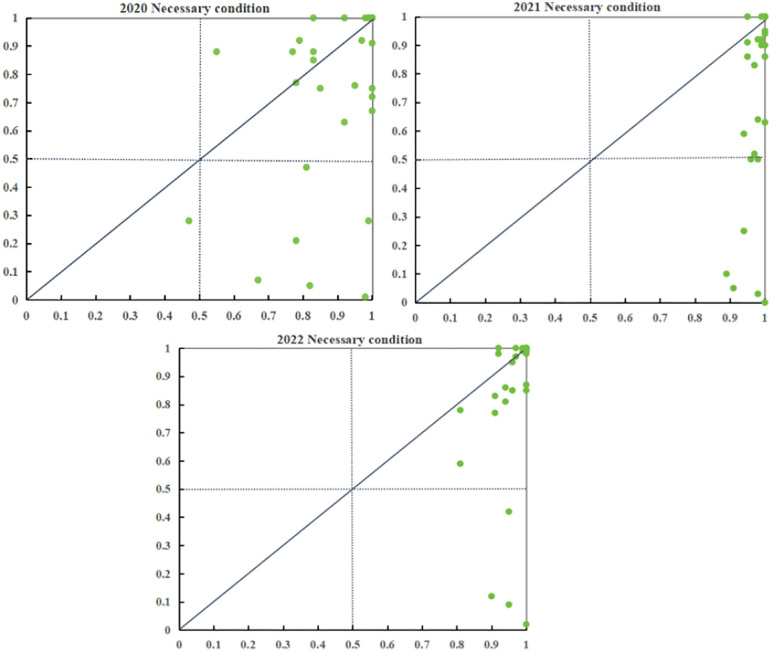
Digital development – Common prosperity necessity test scatter plot.

As shown in Table 5, except for the consistency level of digital development driving common prosperity during the period of 2020–2022 being greater than 0.9 and the coverage rate being greater than 0.5, the other conditions do not have necessary relationships. Further, the XY scatter plot of “Digital development - Common prosperity” for the period 2020–2022 shows that the results do not meet the necessity test, indicating that digital development is not a necessary condition for common prosperity (Fig 5).

**Fig 6:**
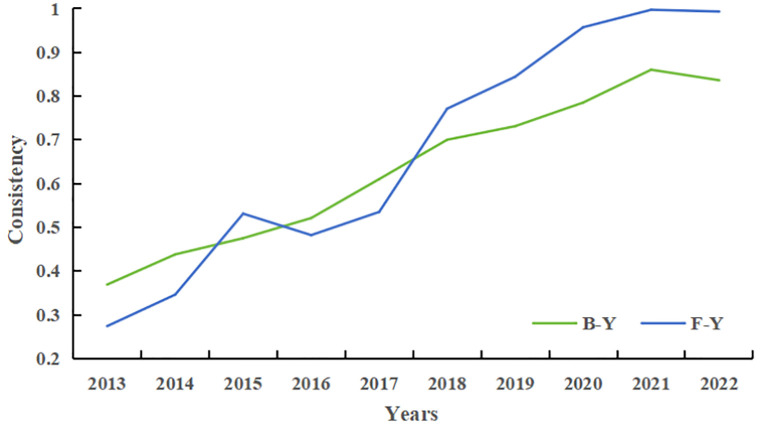
Trend of consistency change.

It is worth noting that during the sample observation period, the importance of laborer awareness and digital development for common prosperity has gradually strengthened, demonstrating the significant potential of digital laborer awareness and digital technology development (Fig 6). In the future development and construction of common prosperity, the province should fully stimulate the potential kinetic energy of digital personnel, achieve industrial digitalization and intelligent transformation, and promote common prosperity.

### 5.2. Configuration adequacy analysis

This paper, based on the relevant QCA theories and the characteristics of the data, selects 0.9 as the consistency value, 0.7 as the PRI value, and 2 as the case frequency to construct the truth table [63]. Then we proceed to the reinforcement standard analysis to eliminate contradictory counterfactual hypotheses. Since existing studies have not reached a unified conclusion regarding the impact of various antecedent conditions on common prosperity, this paper adheres to the principle of caution and makes no pre-assumptions about the direction of the variables. Ultimately, we arrived at three solutions. We distinguished the core conditions from the peripheral conditions based on the nested relationship between the intermediate and simple solutions. Table 6 reports the three types of paths: “Digital intelligence foundation - talent traction type”, “Digital intelligence industry - talent linkage type”, and “Digital intelligence development-talent collaboration type”. Among them, the potential of labourers and the new quality industry are the core variables in the configuration path. The overall consistency of the configuration path is 0.941, and the overall coverage is 0.674. According to Fiss’s [64] research, the minimum standard for consistency level is 0.750. The consistency of individual paths and the overall path is higher than the standard value, indicating that each path can fully explain the development of common prosperity. Digital new quality productivity is the key strategy for advancing common prosperity.

**Table 6:** Configuration adequacy analysis results.

Condition variable	Configuration H1	Configuration H2	Configuration H3
Labourer potential	●	●	●
Labourer consciousness	•	–	•
New quality industry	●	●	●
Digital foundation	●	•	–
Digital industry	–	●	⨂
Digital development	–	–	●
Consistency	0.948	0.950	0.958
PRI	0.936	0.939	0.897
Coverage	0.579	0.619	0.139
Unique coverage	0.022	0.089	0.007
Adjustment distance between groups	0.033	0.036	0.098
Adjustment distance within Group	0.161	0.161	0.155
Total PRI		0.928	
Total consistency		0.941	
Total coverage		0.674	

Note: ● indicates has a core condition, • indicates has an edge condition, ⊗ indicates no core condition, ⨂ indicates no edge condition, and blank indicates presence or absence.

#### 5.2.1. Summary consistency analysis.

(1) Digital intelligence foundation – talent traction type

Configuration H1 (High digital foundation * High new quality industry * High labourer potential * Labourer consciousness) is applicable to provinces with well-developed digital infrastructure. This pathway represents an infrastructure-driven model within the techno-economic paradigm: the diffusion of general-purpose technologies, underpinned by advanced digital infrastructure, lays the technological foundation for broad economic participation and social inclusion. The digital foundation, through extensive linkages and underlying support, ensures that economic outcomes are comprehensively covered and equitably accessible, laying the inclusive foundation for common prosperity. The deep application of artificial intelligence and industrial robots follows the logic of technological progress. It not only reshapes the employment structure and gives rise to new forms of employment, but also enhances workers’ employment awareness and entrepreneurial vitality, promoting the coordinated evolution of human capital and technological innovation, and building an innovation-driven growth mechanism for common prosperity.

A typical example of this configuration is Shanghai. Shanghai has issued a series of policies to support the construction of digital facilities and the application of intelligent technologies. Initiatives such as the creation of new networks, new platforms, new infrastructure, and new intelligence under the category of new infrastructure have lowered the barriers to innovation and entrepreneurship, providing more development opportunities and income channels for small and medium-sized enterprises, individual workers, and residents. At the same time, the application of digital technologies has promoted the equal distribution of public services such as education, healthcare, and government affairs, allowing citizens to share the fruits of economic development and further narrowing the social gap caused by the digital divide. Moreover, Shanghai continuously attracts junior digital professionals and cultivates and exports a mid- to senior-level digital talent backbone. The digital talent cultivation and mobility mechanism enhances workers’ vocational skills and market competitiveness, thereby increasing overall labor value and salary levels. This pathway reflects the supporting role of digital infrastructure, where the synergistic drive of new industries, talent systems, and other elements provides foundational support and policy assurance for the construction of common prosperity.

(2) Digital intelligence industry – talent linkage type

The configuration of H2 (High labourer potential * High new quality industry * High digital foundation * Digital industry) applies to provinces with a high degree of digital industrialization. This pathway shows the industrial restructuring model in the techno-economic paradigm. Here, the integration of digital technologies with the real economy drives structural change and productivity gains, supporting common prosperity. Digital infrastructure acts as a universal platform. By connecting production factors, it accelerates the shift of traditional industries toward digital and intelligent operations, creating digital, intelligent industries. This transformation affects common prosperity in two ways. On the industrial side, digital transformation overcomes the time and space limits of traditional industries. It boosts the resilience and flexibility of the industrial chain, building a stronger system for common prosperity [65]. On the talent side, it helps human capital, industrial needs, and scientific and technological innovation adapt quickly. This enables efficient allocation of resources and supports steady growth and circulation of social wealth.

A typical case of this configuration is Guangdong Province. The province has expedited the implementation of the “Industrial Guidelines for the Guangzhou Artificial Intelligence and Digital Economy Pilot Zone” and the “Several Policies for Promoting Digital Transformation of Manufacturing Industry in Guangzhou”. The aim is to leverage digital technology to drive transformation and upgrading across the manufacturing and service industries, thereby creating more high-skilled jobs and flexible employment opportunities for workers. Furthermore, by developing public infrastructure such as the Hengqin Smart Computing Platform and the 5G Innovation Center, Guangdong has lowered the technological innovation threshold for small and medium-sized enterprises, fostering the transformation of scientific and technological achievements. In addition, Guangdong has established a digital talent hub in the Guangdong-Hong Kong-Macau Greater Bay Area and launched pilot reforms to the talent development system, facilitating dynamic matching of human capital with industrial needs. This has laid a solid foundation for high-quality employment and income growth, enabling workers to share in the development dividends of industrial upgrading and advancing the cause of common prosperity. This path takes digital industry development as the core driving force, jointly providing systematic support for inclusive growth and sustainable sharing mechanisms through the combination of digital infrastructure, artificial intelligence, and human capital.

(3) Digital intelligence development – talent collaboration type

Configuration H3 (High labourer potential * High new quality industry * digital industry * High digital development) is applicable to provinces with a high level of digital development. This path represents a model of deep integration and systematic transformation of the technology-economic paradigm: digital technology is fully integrated into the economic and social system, becoming the core force driving institutional innovation, governance optimization, and the development of common prosperity. The evolution of digital infrastructure and intelligent industries has driven digital productivity, characterized by technological innovation, artificial intelligence, and the digital economy. While driving the transformation and upgrading of digital technology talent, it is also reshaping the social employment structure and creating new digital-based occupations. It not only improves the efficiency and structure of resource allocation but also effectively addresses unbalanced resource distribution and enhances the degree of sharing within the benefiting group. In addition, the digital economy and technological intelligence accelerate economic system reform, giving rise to new models, markets, and driving forces within the economic development system, providing new space for the construction of common prosperity.

A typical case of this configuration is Heilongjiang Province. This province leverages the national enterprise technology center and the national innovation demonstration enterprise to promote the intelligent transformation of traditional industries, thereby creating new occupations such as data analysts and agricultural digital specialists. This provides workers with higher-skilled and more sustainable employment channels, enhancing the possibility of income growth. Moreover, the branches of Heilongjiang Agricultural Bank have continuously advanced digital transformation, developing digital financial products covering rural areas and small and medium-sized enterprises, enabling remote areas and disadvantaged groups to access convenient credit and financial services, effectively alleviating the issue of uneven resource distribution between regions and between urban and rural areas. Additionally, the province attaches great importance to the construction of digital talent teams, actively establishing digital skills talent bases, and launching targeted vocational skills programs to help workers in traditional industries and college graduates adapt to the demands of digital economic development, enhancing their employment competitiveness and income-generating capabilities in the digital age. This approach takes digital development as the core driving force, collaborating with artificial intelligence innovation, digital infrastructure, and talent system construction, promoting the transformation of technological dividends into inclusive growth outcomes, and providing a replicable, practical model for common prosperity.

In summary, the three types of pathways achieve common prosperity in distinct yet convergent ways, with both horizontal and vertical comparability as well as dynamic interactions among them. From the perspective of horizontal development, the coverage of each path is relatively low. This indicates that the dependence between different provinces in achieving high levels of common prosperity development is weak, and there may be a substitution relationship among the paths. Specifically, configurations H1 and H2, under the common conditions of labourer potential, new quality industry, and digital foundation, exhibit mutual substitution between labourer consciousness and the digital industry, forming second-order equivalent configurations. Based on the labourer’s potential, labourer awareness, and the emergence of a new quality industry, the configuration of H1 and H3 allows for substituting non-digital industries and high digital development combinations with high digital foundations, thereby forming an equivalent configuration. From a longitudinal perspective, in the early and middle stages of digital new quality productivity development, digital new quality productivity, represented by digital foundation and the digital industry, first emerges as the dominant pathway driving progress toward common prosperity. As digital new quality productivity continues to expand, the “digital intelligence development - talent collaboration” path, formed by the combination of the digital economy, artificial intelligence, inclusive finance, and digital talents, is gradually playing a supporting role in the construction of common prosperity across various provinces.

#### 5.2.2. Between results analysis.

**Fig 7:**
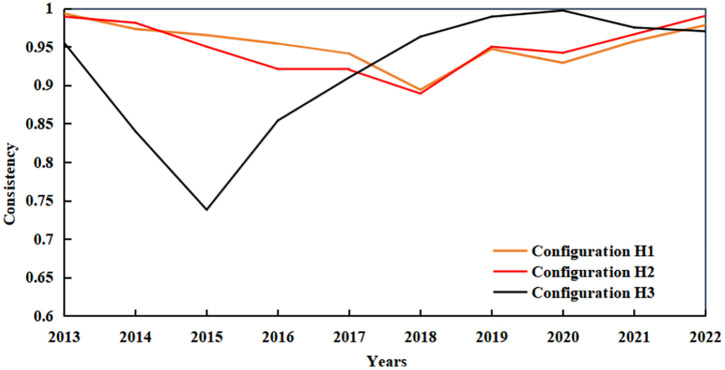
Inter-group consistency changes.

As shown in Table 6, the consistency adjustment distance between groups is less than 0.2, and there is no obvious time effect. From the perspective of the time dimension, the paths of configurations H1 and H2 are relatively stable, and the path changes exhibit slight fluctuations in amplitude. The configuration H3 shows a “U” shaped development trend starting from 2015 (Fig 7). From the perspective of path evolution, the digital new quality productivity has undergone a progressive development process, from “digital intelligence foundation” to “digital intelligence industry,” and ultimately to “digital intelligence development.” Among them, the “digital intelligence foundation-talent traction” in the first stage represents the infrastructure-driven model of the technological economic paradigm, which is the main force driving the construction of common prosperity; the “digital intelligence industry-talent linkage” in the second stage represents the industrial reconfiguration model of the technological economic paradigm, and it gradually plays a core role in the construction of common prosperity; the “digital intelligence development-talent collaboration” in the third stage represents the deeply integrated and systematic transformation model of the diffusion of the technological paradigm, and it is the dominant mode for facilitating the construction of common prosperity.

#### 5.2.3. Within results analysis.

**Fig 8:**
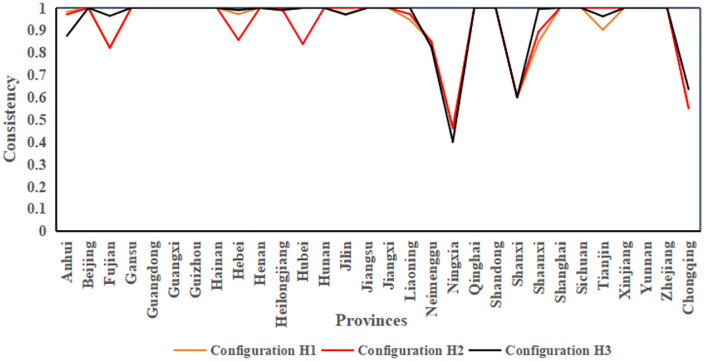
Within-group consistency changes.

As shown in Table 6, the intra-group consistency adjustment distance is less than 0.2, and there is no significant cross-interval effect. In some regions of the central and western parts (such as Fujian, Ningxia, Shanxi, and Chongqing), the configuration path has a relatively weak explanatory power for the development of common prosperity and does not represent a positive deviation [37] (Fig 8). The reason why the consistency level of the configuration path shows a collective decline in certain regions might be that the digital new quality productivity driving the common prosperity development exhibit heterogeneity, specifically manifested as “east-west differences” and “east-central differences” [47,66].

### 5.3. Robustness test

The QCA method, due to potential threats in its parameter settings and model design, leads to sensitivity and randomness in research outcomes. Therefore, a robustness test is necessary for it. Based on this, this paper conducts robustness tests by adjusting the case frequency threshold, the consistency level, and the PRI threshold in three ways [67,68]. Based on each path and the set relationship of the core variables within the paths, determine the robustness test results. After the inspection, it was found that the configuration path did not change significantly, which met the robustness test standards, indicating that the research results were reliable. Due to space limitations, the results of the robustness test are not presented in this article.

## 6. Discussion

This article concludes that the overall level of common prosperity in China rose from 2013 to 2022, with a distinct regional distribution pattern characterized by “higher development in the eastern regions and relatively lower levels in the western regions.” This result is consistent with prior research findings. As noted by Ma et al. [69], from 2010 to 2020, the degree of common prosperity in China has shown a rising trend. Furthermore, the eastern region demonstrates the highest level of common prosperity, whereas the western region exhibits the lowest. Zhao et al. [26] assessed the regional development of common prosperity and found that levels in the eastern region were significantly higher than those in the western region. These studies have revealed the uneven characteristics of common prosperity development in our country, thereby providing a focused direction for leveraging digital new quality productivity to promote regional common prosperity.

This study shows that no single factor alone determines the path of common prosperity development. Instead, several factors interact to create different ways to achieve common prosperity. This view aligns with the research by Cheng et al. [51], who argue that multiple factors influence common prosperity at various levels, including technology, organization, and environment. These factors interact in complex ways to form three main paths. Thus, it is important to use a holistic approach to study how these factors interact and work together, so we can identify possible paths that inform policies for achieving common prosperity across different contexts.

Current research on common prosperity predominantly examines the isolated effects of individual factors – such as technology, industry, and organizational elements – on common prosperity, while largely overlooking their interconnected impacts and systemic interactions. These quantitative studies lack consideration of the complex system theory and the intricate relationships among various factors [64]. Although a small number of studies have examined the synergistic and interactive effects among elements, they fail to analyze the configuration paths from a temporal perspective, thereby failing to adequately capture the complexity and dynamic nature of path development [59]. Therefore, building upon existing research, this paper not only examines the multifaceted impact of complex antecedent conditions associated with the digital new quality productivity on common prosperity, but also investigates the dynamic evolution mechanism of the configuration path of common prosperity from a temporal perspective. This contributes to a deeper understanding of the complex driving mechanisms underlying the achievement of common prosperity in the digital era and offers a reference framework for promoting common prosperity through the development of digital new quality productivity.

## 7. Research conclusion and policy implication

Digital new quality productivity signify the progressive development of advanced productive forces and serve as a crucial mechanism for advancing robust common prosperity. This paper employs panel data from 30 provincial regions in China, integrating static development evaluation with dynamic resource allocation analysis to comprehensively assess the current state and evolutionary trends of common prosperity in the country. Furthermore, it explores the underlying mechanism through which digital new quality productivity promotes the development of common prosperity. The key findings are as follows:

(1) The level of common prosperity in our country has generally shown an upward trend. Compared to other regions, the eastern region exhibits a higher degree of common prosperity. The development of common prosperity in the central and western regions is relatively comparable, while progress in the northeastern region remains uneven. The overall development of common prosperity exhibits the characteristics of “leading in the East, rising in the Central region, development in the West, and revitalization in the Northeast.”
(2) The digital new quality productivity conditions in each province enhance common prosperity through a “divergent paths converging towards the same goal” approach, with no single factor being sufficient to support the development of common prosperity. There are three types of driving paths: the “digital intelligence foundation-talent traction type,” “digital intelligence industry-talent linkage type,” and “digital intelligence development-talent collaboration type.” There is a certain development pattern in the configuration paths of common prosperity. From a temporal perspective, the path of digital new quality productivity has evolved from “digital intelligent foundation” to “digital intelligent industry” and then to “digital intelligent development.” From a spatial perspective, the impact of digital new quality productivity on common prosperity shows a nonlinear influence. The configurational differences in regional explanations mainly manifest as the “East-Central disparity” and “East-West disparity.”

The above research demonstrates that digital new quality productivity significantly enables common prosperity, serving as a crucial strategy for promoting both balanced development and high-quality advancement of common prosperity. Therefore, this paper proposes the following potential policy recommendations:

(1) The state should implement differentiated digital new quality productivity strategies, tailored to regional disparities in common prosperity. For the eastern region, local governments should shift the policy focus from “scale expansion” to “quality improvement,” transforming digital dividends into inclusive public services by establishing cross-border data pilot zones and opening computing power markets, thus driving common prosperity through inclusive growth. For the central, western, and northeastern regions, local governments should focus on “addressing weaknesses and enhancing resilience.” By relying on national-level computing power hubs and the East-West cooperation fund, they should guide the synchronized development of digital infrastructure, specialized talent, and data elements, integrating digital new quality productivity into rural revitalization and industrial upgrading. This will help narrow the digital divide and development gap between regions, laying a solid foundation for advancing common prosperity.
(2) This article proposes three types of configuration paths, through which the government can guide provinces to choose a development model for common prosperity based on their own digital resources and talent advantages. For regions with relatively well-developed digital infrastructure but insufficient talent reserves, local governments should focus on building mechanisms to attract and cultivate digital talent. By implementing policies such as special subsidies, tax incentives, and support for research platforms, they can attract high-end digital talent and leverage the talent dividend to enhance the effectiveness of digital infrastructure, thus contributing to common prosperity. For regions with a solid industrial foundation but lagging digital transformation, local governments should increase the penetration of digital technologies into traditional industries. This can be achieved by establishing platforms for industry-university-research cooperation, setting up transformation-specific funds, and promoting the synergy between digital industries and local industries. This approach will expand income channels through industrial linkage, supporting common prosperity. For regions with a certain foundation in digital technologies, industries, and talent, local governments should focus on promoting deep collaboration among the three. By creating a virtuous interaction system between “technology-industry-talent,” they can strengthen the multi-dimensional driving effect of digital new quality productivity on common prosperity.
(3) Establish differentiated configuration pathway monitoring and cross-domain flow mechanisms. To address temporal discrepancies in configuration pathways, the government can establish a “Digital new quality productivity - Common prosperity” monitoring platform that enables real-time monitoring and dynamic analysis of the sequential combinations of key elements, such as data, computing power, and algorithms. By setting up an element flow evaluation system and a cross-temporal collaborative regulation mechanism, the government can precisely identify imbalances in factor allocation and develop phased optimization strategies. This will ensure that the spillover effects of digital new quality productivity accurately serve the goals of increasing residents’ income, narrowing the urban-rural gap, and equalizing public services over time. Regarding differences in spatial development and configuration paths, the government should establish a cross-regional flow system for data resources, digital technologies, and digital talent. This will facilitate the orderly transfer of high-quality digital elements from the eastern region to the central, western, and northeastern regions, providing digital impetus for the common prosperity development of underdeveloped areas.

The following limitations and shortcomings are present in this study. Firstly, this study establishes a digital new quality productivity index system encompassing three dimensions: digital labourer, object of digital labour, and digital means of labour, based on prior research. It encompasses three facets of production factors, yet there may be relatively limited indicators. In the future, it is essential to systematically improve the index system for digital new quality productivity to facilitate the advancement of common prosperity. Secondly, this study is a macro analysis utilising China’s provincial panel data, which does not further dissect the influence of digital new quality productivity on promoting common wealth. It can be further explored in the future by dividing the sample data at the prefecture-level city levels. Finally, this paper supports the research findings through robustness tests; however, it may not fully eliminate the endogeneity issue arising from unobservable factors. Future research could explore novel methodological approaches that integrate causal inference techniques, such as instrumental variable methods, with dynamic Qualitative Comparative Analysis (QCA) to further validate the causal inferences drawn from this study.

## Supporting information

S1 File Raw_images. (ZIP)

S2 File Supporting information--origin data. (ZIP)
